# Supplementary material for: Increase of Clostridioides difficile PCR ribotype 002 infection with decreased disease severity in hospitalized patients in the Netherlands
Source: Microbiol Spectr. 2025 Aug 19;13(10):e01446-25. doi: 10.1128/spectrum.01446-25 (PMC12502521; doi:10.1128/spectrum.01446-25)
Supplement: Fig. S1 — Annual incidence of RT002 CDI. [file spectrum.01446-25-s0001.pdf]

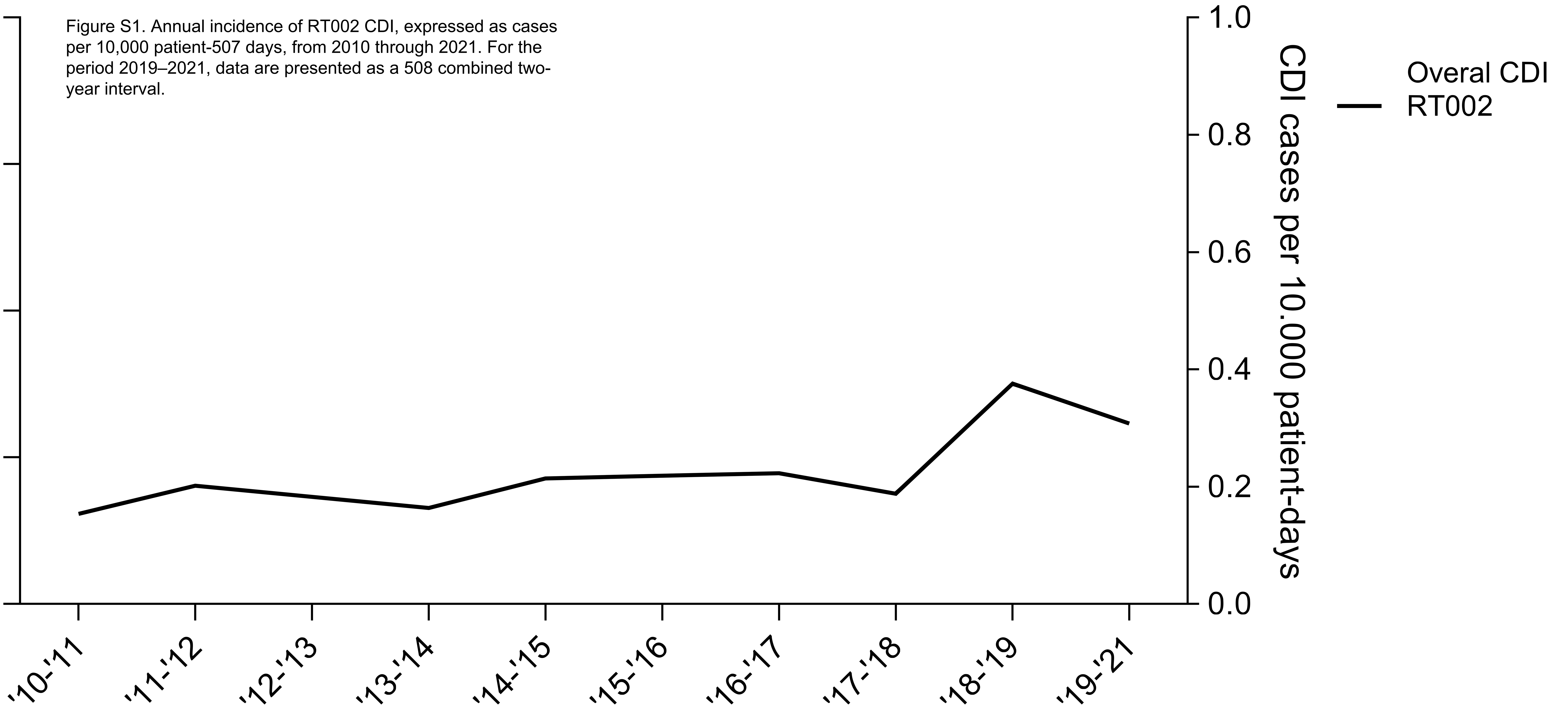

Figure S1. Annual incidence of RT002 CDI, expressed as cases per 10,000 patient-507 days, from 2010 through 2021. For the period 2019–2021, data are presented as a 508 combined two-year interval.
